# Foodborne pathogens in Africa: Understanding *Cronobacter sakazakii*

**DOI:** 10.1002/puh2.53

**Published:** 2023-01-11

**Authors:** Ifeanyi Michael Mazi, Helen Onyeaka, Nnabueze Darlington Nnaji

**Affiliations:** ^1^ Department of Microbiology, Faculty of Life Sciences University of Benin Benin City Nigeria; ^2^ School of Chemical Engineering University of Birmingham Birmingham UK; ^3^ Department of Microbiology, Faculty of Biological Sciences University of Nigeria Nsukka Nigeria

**Keywords:** *Cronobacter sakazakii* contamination, detective methods, epidemiological report, food process controls

## Abstract

*Cronobacter sakazakii* is an emerging and opportunistic foodborne pathogen that causes severe infantile diseases, including meningitis, necrotizing enterocolitis, and septicemia. It has been reported in numerous countries around the world, including those in Africa. Although it has been isolated from food, environmental and clinical samples across Africa, the most implicated source of the *C. sakazakii* infection outbreaks across the globe has been the consumption of contaminated powdered infant formula. *Cronobacter* has many unique characteristics that contribute to its survival in harsh environments and transmission along the food chain from production to consumption. A potential foodborne disease outbreak caused by *C. sakazakii* can increase the overall foodborne disease burden and hinder any progress in managing the overly strained public health situation in Africa. This article presents an insight into the occurrence and prevalence of *C. sakazakii* infection in Africa's food environment, pointing out the transmission route along the food chain and its accompanying food safety concerns. This paper advocates for strict compliance with food safety and control measures to prevent its spread in African countries.

## Graphical abstract

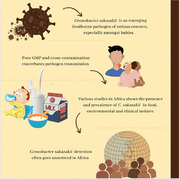

## INTRODUCTION

*Cronobacter sakazakii*, an emerging opportunistic foodborne pathogen [1, 2, 3], is associated with rare but severe infantile infections such as meningitis, necrotizing enterocolitis, and septicemia. It has been reported to affect most infants with immunocompromised systems or low‐birth weights, and the elderly [2, 4, 5, 6, 7]. Neonatal infections associated with *C. sakazakii* have been reported to have a mortality rate as high as 80%, often leading to irreversible neurological disorders for survivors [5, 8].

The natural habitat and the primary host, including its genomic information and comparative sequence analysis, virulence factors/pathogenicity, and strain genetic diversity remain largely unidentified [6, 9]. *C. sakazakii* is a pathogenic bacterium that has been isolated from both food and the environment as well as clinical isolates [6]. As an environmental microorganism, it has been isolated from powdered infant food (PIF) manufacturing plants traceable to a breach in good manufacturing practice (GMP) in the food processing facilities [2], as well as in water and soil [1]. Contaminated household kitchenware such as spoons, blenders, and infant bottles are also reported source where *C. sakazakii* have been isolated [10, 11]. *C. sakazakii*, as a foodborne pathogen, is implicated in several low‐moisture food products, including contaminated PIF [6], cereal, dried herbs, pasta, grains, fermented bread, meat and meat products, mixed salad vegetables, cheese, and fruits [4, 9]. However, the most epidemiologically implicated food source has been contaminated PIF [12].

*C. sakazakii* is a genomically heterogeneous, peritrichously flagellated, motile, Gram‐negative, rod‐shaped, non‐spore‐forming, facultative anaerobic coliform bacterium within the family Enterobacteriaceae, and genus *Cronobacter* [3]. Formerly referred to as *Enterobacter sakazakii*, improved microbiological identification techniques involving a detailed polyphasic taxonomical approach, 16S rDNA sequencing, multilocus sequence typing (MLST), and biotyping [6, 9], it was considered a genus of its own within the Enterobacteriaceae family. *C. sakazakii, Cronobacter turicensis, Cronobacter malonaticus, Cronobacter dublinensis, Cronobacter muytjensii, Cronobacter condimenti*, and *Cronobacter universalis* are the seven species of the genus *Cronobacter* [13]. Table 1 shows the biochemical characteristics of *C. sakazakii*.

**TABLE 1:** The biochemical test of *Cronobacter sakazakii*

Biochemical test	Result
Indole	−
Methyl red test	−
Acetoin production (VP test)	+
Citrate utilization	+
Phenylpyruvic acid production	±
Lysine decarboxylase	−
Ornithine	−
Arginine hydrolyzation	+
H2S production	−
Lactose	+
Trypticase soy agar at 25°C	Yellow pigmented
DNase test on toluidine blue agar (36°C, 7 days)	+
Catalase	+
Oxidase	−
Urease	−
Growth in KCN	+
Tween 80 esterase production	+
d‐Sorbitol	−
Phosphoamidase activity	−
α‐Glucosidase activity	+

*Source*: [6].

Epidemiologically, only contaminated PIF has been linked to infantile diseases caused by *Cronobacter* [14]. However, PIF contamination can occur during the production stage for several reasons, such as poor GMP and contaminated equipment [2]. Owing to its high food safety risk and severity of *C. sakazakii* infections, especially for infants, the International Commission on Microbiological Specification for Foods in 2002 categorized *Cronobacter* spp. as “a serious hazard for restricted populations, life‐threatening or with significant chronic sequelae over a long duration” [2, 15].

*C. sakazakii* is a unique organism of concern in the food industry because of its exhibition of various characteristic features that help the organism live under unfavorable conditions, thus contributing to its survival [16]. These features include the formation of biofilm [17, 18]; the ability to withstand critical food control steps during food production such as osmotic stress, thermal treatment, pH, detergents, starvation, disinfectants, antibiotics, and sanitizers. In addition, the organism also possesses the ability to significantly resist high temperatures (the minimum, maximum, and optimum temperatures for growth are 6, 45, and 37–43°C) [3, 19] and desiccation that allows it to stay up to 2–5 years in an encapsulated form in dehydrated food products (with water activity range from 0.30 to 0.83) [20].

## EPIDEMIOLOGY AND VIRULENCE FACTORS OF *CRONOBACTER SAKAZAKII*

### Epidemiology

According to the World Health Organization and the Food and Agriculture Organization, cases of *Cronobacter* infections amongst infants and young children were compiled by international organizations and private firms such as the International Special Dietary Foods Industries, International Formula Council, the US Center for Disease Control, Nestle, UCD, as well as several authors, although the reports were never published. Based on their findings, approximately 120 *Cronobacter* infections were recorded among newborns and young children under three globally [21]. However, the sporadic nature of the documented cases of *Cronobacter* infection in children makes it challenging to ascertain the source of infection during an outbreak, causing researchers to depend on microbiological testing of suspect vehicles instead of epidemiological methods in determining the source of infection. Worthy of note is that (1) such a testing method depends on the retention of potential vehicles in sufficient amounts to achieve valid testing while storing them properly, and (2) the test may not be the best approach, especially for large‐scale foodborne outbreaks.

Based on the current surveillance systems across the globe, available data shows that there is no active surveillance system for diseases caused by *E. sakazakii (Cronobacter* spp.), implying that most national foodborne disease surveillance centers of the world are yet to identify cases of *C. sakazakii* infection. Because *C. sakazakii* infection is rare, most sites where *Cronobacter* spp. disease has been reported have very low populations, and several years of monitoring/surveillance will be needed to develop a valid incidence estimate across affected populations [21]. Many countries declared that their outbreak management system and/or foodborne disease surveillance system could detect an outbreak of *E. sakazakii* (*Cronobacter* spp.) infection. Yet, for the most part, reported cases of *Cronobacter* spp. were through outbreaks or voluntary passive reporting rather than through the national reporting systems for foodborne diseases [21]. Through voluntary passive reporting, unpublished cases of *Cronobacter* spp. have been made public. Although several countries have laboratory‐based surveillance systems capable of detecting *Cronobacter* spp. alongside other diseases like nosocomial infections, bacteremia, or antibiotic resistance infections, these laboratory data are often insufficient to investigate and determine the exposure factors. According to available evidence, newborn babies are more likely to fall ill and/or die from *Cronobacter‐related* diseases. As exposure is assumed unusual and reporting is not mandatory, community‐based epidemics among older children are far less likely to be detected than hospital‐based outbreaks amongst very young babies [21, 22].

Generally, in less developed nations, including Africa, the impact of *Cronobacter* spp. often goes unnoticed [23]. This is corroborated by findings from Ethiopia that the overall burden of *Cronobacter* is grossly underreported and, to some degree, considered non‐reportable compared to the significant number of other foodborne pathogens reported [3]. Furthermore, the available data on *Cronobacter* spp. prevalence in follow‐up formula products for babies aged 6–11 months is very scarce. This scarcity is because only a few national regulatory bodies have been able to define the Microbiological Specifications for *Cronobacter* spp. in these items [21]. Despite the increase in the number of carefully documented cases of *C. sakazakii* (most infections in infants), this number is significantly low compared to many other infectious diseases. Currently, the actual burden of *Cronobacter* spp., in Africa or worldwide, cannot be determined as no effort has been made to estimate the factors that might be used to determine the overall burden. The capacity of environmental, clinical, and food laboratories to successfully identify *C. sakazakii* infections remains a major limitation in their diagnosis. Although significant advancement has been made with the identification methodology, there is still the need for increased awareness and capacity building, as with other emerging infections [21].

### Virulence factors

*Cronobacter* has several special characteristics that contribute to its ability to live in a harsh environment, which may be advantageous to the organism when internalized in a host [23]. Therefore, in order to understand the pathogenesis and host–pathogen interaction of pathogenic microbes, clear identification of the bacterial virulence factors is critical, especially as such knowledge will aid in the development of appropriate drug and vaccine that targets the pathogen's defense mechanism [24]. Although the virulence factors and pathogenic mechanism are yet to be clearly understood, it is known that *C. sakazakii* has the *O*‐antigen, produces proteolytic enzyme and siderophores, exhibits curli expression, bacterial cell surface hydrophobicity, congo red binding, hemolytic activity, and hemagglutination, all of which are important virulence factors for bacterial pathogens [25, 26].

The successful colonization of the host gastrointestinal tract by *C. sakazakii* to cause a disease involves a multifactorial process and the activation of selective virulent genes [27]. As part of its pathogenic mechanism, *C. sakazakii* has an outer membrane protein A (OmpA) (a fibronectin‐binding protein for host cell adhesion and attachment, growth, differentiation, and movement) that aids in its colonization of the host gastrointestinal tract [28, 29] and subsequent invasion of the intestinal epithelial and human endothelial microvascular brain cells (HBMEC) [30] within 60 min [31], thus crossing into the bloodstream where it causes meningitis [29, 32]. Also, another outer membrane protein (OmpX) plays an essential role in the bacteria attack on the host Hep‐2 cells and Caco‐2 epithelial cells [33]. The entering of the OmpA and OmpX into the blood–brain barrier results in brain cell necrosis, although the mechanism of action is yet to be fully understood [34]. A deficiency of the OmpA protein in the bacteria has been found to decrease HBMEC invasion by 83% [28]. A study showed that the outer membrane protein W (OmpW) contributes to *C. sakazakii* cells’ survival under NaCl stress. OmpW is an important outer membrane protein that plays a vital role in the response of pathogenic microorganisms to osmotic stress or salt regulation. However, the roles of OmpW under NaCl stress are dependent on the *Cronobacter* species or prevailing environmental conditions [1].

Additionally, certain strains of *C. sakazakii* have the ability to persist or replicate in macrophages for close to 48 h [21]. Different strains of *C. sakazakii* have been shown to survive differently in macrophages depending on the presence of putative sod genes that encode a superoxide dismutase [35]. As part of their virulence factor, the organism's flagellum helps activate proinflammatory cytokines in macrophages using several flagellation genes [36, 37]. It also serves as an essential tool for the formation of biofilm and host cell attachment [38]. With regards to biofilm formation, *C. sakazakii* strains have been reported to release molecules of quorum sensing (QS) referred to as *N*‐acyl‐l‐homoserine lactones (AHLs) [18]. The successful inhibition of the AHL‐mediated QS has proven to be a novel target for inhibiting the formation of biofilm by *Cronobacter* spp. [16].

Other virulence factors exhibited by *Cronobacter* spp. include the ferric decipher transport framework that is responsible for the procurement of iron [39]; the formation of capsule (i.e., encapsulation) that enables the organism to settle and easily attach to plant surfaces [40]; and the formation of *O*‐antigen (*O*‐polysaccharide, a lipopolysaccharide factor on the external layer of Gram‐negative bacteria) [41].

*C. sakazakii* is resistant to a number of antibiotics, including gentamicin, vancomycin, chloramphenicol, ampicillin, nitrofurantoin, penicillin G, imipenem, tetracycline, and doxycycline [25]. Although WHO reports point out that *C. sakazakii* isolates from environmental, food, and clinical food sources appear to differ in pathogenicity, current shreds of clinical evidence support the fact that all strains of *Cronobacter* genus, except *C. condimenti* with no recorded clinical evidence [3], are pathogenic and pose a great risk to infant and neonatal health [21].

## PREVALENCE AND OCCURRENCE OF *CRONOBACTER SAKAZAKII* IN AFRICAN COUNTRIES

Despite global research to determine *C. sakazakii* prevalence, its epidemiology remains incomplete and poorly understood [42]. Given that *C. sakazakii* is ubiquitous in animate (man, animals) and inanimate (soil, water, plants) environments, it is not unusual that *C. sakazakii* has been isolated from a variety of foods as well as animal and vegetable sources of food products [43], though little is known about *Cronobacter* existence in their various environments [44]. The most common food implicated in *Cronobacter* infection globally is PIF [12].

The prevalence of *C. sakazakii* in South Africa is similar to that observed globally. In 2008, 14% of the isolates obtained from South African newborn formula milk and the processing environment tested positive for *C. sakazakii* [45]. This result corroborated a previous study that showed that 18% of South African infant formula milk and other baby food products were contaminated with *C. sakazakii* [46].

In North Africa, several studies on *Cronobacter* infection have been done. A 2013 study in Egypt revealed that *C. sakazakii* was present in the analyzed cheese, ice cream, and yoghurt samples. The isolation method employed chromogenic medium and traditional culture media [9]. It was discovered that *C. sakazakii* was a significant cause of neonatal sepsis amongst preterm infants in North Africa. The identified means of bacterial infection were contaminated PIF, herbs, and water [22]. In another research, 15% of the analyzed ground beef and beef burger samples sold in Egypt tested positive for *C. sakazakii*. Their results further established new epidemiological evidence that these pathogens are widespread in meat products sold in Egypt. However, this bacterium's epidemiology remains incomplete and poorly described [42].

In West Africa, *C. sakazakii* was isolated from 13% of the 185 samples analyzed to determine the occurrence of *Cronobacter* spp. in PIFs sold in Abidjan, Ivory Coast. The study proved to be the first published work on the contamination of PIF with *C. sakazakii* from Abidjan [47]. Also, the presence of *C. sakazakii* isolate from pharmaceutical industries wastewaters confirmed the prevalence of *C. sakazakii* in Nigeria's ecosystem [44].

Conclusively, underreporting is attributable to the apparent absence of foodborne diseases caused by *Cronobacter* amongst malnourished children who are also vulnerable to severe infections in Africa [48]. This is based on the argument that in the biggest and longest running holistic surveillance carried out for invasive bacterial infections in children and infants in sub‐Saharan Africa, just a few instances of invasive *Cronobacter* infection were reported. Of the 65,426 samples collected between 1998 and 2013, 3953 were blood and cerebrospinal fluid cultures, of which only 60 tested positive and were identified as *Cronobacter* species. Worthy of note is that 39 of those 60 cultures identified as *Cronobacter* species were samples obtained from newborns. However, employing an automated culture system to validate the isolated microorganisms further using a biochemical profile testing based on US Food & Drug Administration Bacteriological Analytical Manual, only two isolates, both from blood cultures, were confirmed to be consistent with *Cronobacter*. Their study took advantage of the continuous, systematic surveillance research carried out in Kenya by the Kenya Medical Research Institute and the Wellcome Trust Research Programme that lasted from 1998 to 2013 [48].

## TRANSMISSION ROUTES OF *CRONOBACTER SAKAZAKII* INTO THE FOOD CHAIN AND FOOD SAFETY CONCERNS

Clinical presentations of foodborne diseases in newborns have been associated with pathogen‐contaminated PIF, particularly *C. sakazakii* [49, 50, 51]. Food contamination by *C. sakazakii* in PIF has been reported [52, 53, 54], and extrinsic contamination during PIF handling is thought to induce bacterial infection in infants [50]; however, the transmission mechanism is not entirely known. Research has concentrated on food contamination by *C. sakazakii* during manufacture or from raw materials (i.e., intrinsic contamination) [55].

Environmental health practices related to food preparation have been attributed to foodborne illnesses. *Cronobacter* infection has also been connected to unhygienic practices (extrinsic contamination source) by caregivers while preparing PIF [56].

Cross‐contamination from the caregiver to spoon to PIF can be a direct pathway for the exposure to *C. sakazakii* from caregiver to infants; research is limited to observational and surveys studies that do not assess actual risk [49, 57]. Caregivers, and bacterial reservoirs [58], facilitate the transmission of the pathogen to foods through direct or indirect contact. Feeding utensils, including blenders, bottles, and spoons, have been connected to *Cronobacter* infection when contaminated utensils are used in mixing the formula [59]. However, the simulation of PIF handling practices and cross‐contamination of ground foods for the investigation of extrinsic contamination has not been reported [49].

*Cronobacter* is most commonly transmitted to babies through contaminated PIF from opened containers; it can also be transmitted to humans when manufacturers use contaminated raw materials in the manufacturing or when the formula powder comes in contact with a contaminated surface. *Cronobacter* can live in water and on surfaces in the home, including a kitchen sink or counter. Therefore, the formula powder is likely to cross‐contaminate at home after its container has been opened if not properly handled [59].

The pathogen has been reported in wheat and rice plants during the tillering, filling, and mature stages, in water from paddy fields, soil from rice, and soil from wheat flour milling plants [60]. Subtyping using pulsed‐field gel electrophoresis revealed that some strains shared a common profile, implying the widespread presence of *Cronobacter* in the environment, possible cross‐contamination, and transmission routes in processing.

*Cronobacter* spp. has been isolated from dry milk powder, infant formulas, milk and milk‐related beverages, candy and chocolate, and cereals [61]. Additionally, research has been conducted to investigate the prevalence and distribution of *C. sakazakii* in a powdered milk manufacturing plant. In the spray‐drying area, seven pulsed‐field gel electrophoresis types were identified, which supposedly gained entrance into the plant via an improperly controlled roller shutter and a process air aperture. Also, both spray‐drying towers’ textile filters for exhaust air were identified as the pathogen internal reservoirs [62].

*Cronobacter* spp. causes septicemia, necrotizing enterocolitis, neonatal meningitis, and sepsis in infants [22], with a 40%–80% mortality rate [63]. PIF has been identified as the primary mode of transmission. Infants have also become ill due to *Cronobacter* bacteria that grew on breastmilk pump parts that were not adequately cleaned. *Cronobacter* infection was found in families whose staple foods were made from *Cronobacter*‐contaminated wheat flour [60]. *Cronobacter* transmission from human to human is unknown, but there is a possibility because other types of bacteria can be transmitted from one person to another [59].

## MOLECULAR DIAGNOSIS OF *CRONOBACTER SAKAZAKII*

Apart from the conventional screening and identifying *C. sakazakii*, new rapid molecular assay tools have been developed over the years [27]. Some molecular diagnostic methods of detecting *Cronobacter* through specific targets and recognition of the pathogen nucleic acid include the polymerase chain reaction (PCR) method; real‐time PCR; multiplex PCR; repetitive element sequence‐based PCR; PCR‐restriction fragment length polymorphism; pulsed‐field gel electrophoresis; MLST; multilocus sequence analysis (MLSA); multiple‐locus variable number tandem repeat analysis, duplex PCR in mix with slender electrophoresis–laser incited fluorescence locator; molecular *O*‐antigen typing technique; fluorescence in situ hybridization technique; and so on. These molecular methods can detect specific target genes of the pathogen, thus fast‐tracking the identification of the pathogen and tracing the sources of infection [27]. For example, the successful phylogenetic analysis of *Cronobacter* isolates obtained from various environmental and food sources in South Africa was achieved using MLSA of the pathogen's strains based on two genes, 16S rRNA and rpoA [64]. The 16S rRNA gene‐based PCR identification system is a reliable tool for accurately identifying *C. sakazakii* isolates phylogenetically [65]. Consequently, molecular tools can immensely aid epidemiological surveillance and investigation of *Cronobacter* infections and other foodborne diseases in Africa.

## WAYS TO ADDRESS TRANSMISSION OF *CRONOBACTER SAKAZAKII* ALONG THE FOOD PRODUCTION CHAIN

The occurrence of *C. sakazakii* in the food chain is a serious microbiological public health challenge [12]. Food contamination by *C. sakazakii* may come from non‐heated ingredients, contamination from the processing environment, or contamination post‐thermal processing.

*C. sakazakii* transmission can be prevented during food production; therefore, using appropriate microbiological procedures to avoid post‐process contamination will have a positive impact. Standardized analytical procedures are required to ensure product safety [66]. Hazard analysis and critical control point (HACCP), GMP guidelines, and efficient environmental monitoring systems should be developed to control the risk of *Cronobacter* contamination in the food production supply chain, starting from raw materials to finished products [67].

Manufacturers have acknowledged the importance of HACCP and good hygienic practice (GHP) in controlling microorganisms. Raw material quality, sifter screens, liquid and air filters, pasteurization, storage temperatures, and metal/magnets detectors are all critical control points that must be addressed. In addition, the heat treatments used in food manufacturing should be effective enough to inactivate *C. sakazakii* [12].

### Raw material selection

A variety of factors must be examined to guarantee that raw materials used in food production are microbiologically suitable: The possibility of occurrence of *Cronobacter* in ingredients—some are thought to be at high risk of harboring the microbe. In contrast, others are believed to be at low risk [68]. Supplier selection is based on tight requirements (e.g., control measures that are appropriate, verification and release procedures in place, and GHPs) [12]. Testing of raw materials to ensure the efficiency of the steps is mentioned above. Because “raw” material testing alone cannot ensure adherence to the industry's high‐quality standards, manufacturers using these procedures must maintain strong ties with their suppliers of “raw” material and demand strict compliance with HACCP and GMPs.

### Heat treatment

Heat treatment may be a practical approach for removing *C. sakazakii* from abiotic and biotic surfaces. Elevated heat could be used to physically decontaminate important surfaces connected to the preparation and production of foods. It could be used as a processing aid to ensure product safety by using high‐temperature short‐time pasteurization. Temperatures of 70°C or higher were frequently required for *C. sakazakii* decontamination [3].

Other research, however, implies that the organism's osmotolerance may be more relevant in this latter area [19]. The osmotolerant ability of these microorganisms may enhance their chance of becoming much more dominant in the environment, raising the risk and chances of food contamination during post‐processing. The use of typical pasteurization techniques has proven to inactivate *C. sakazakii* effectively [69].

### Post‐processing and packaging

The higher heat resistance of vegetative cells in dry food products is a challenge that must be addressed by evaluating prospective treatments for inactivating microbial pathogens in food [12]. Sterilizing finished products in their dry state in sachets or cans appears to be only conceivable via irradiation. However, because of the organoleptic deterioration of the product, the method does not appear viable with the doses needed to inactivate *C. sakazakii* in dry conditions. Other technologies like magnetic fields and ultrahigh pressure could be viable candidates.

### High‐pressure processing

High‐pressure processing (HPP) is a nonthermal method of inactivating germs by pressuring a packed food in a water‐filled enclosed chamber for a brief period [70]. The use of HPP has the advantage of preserving the freshness, flavor, color, and physical features of foods while causing minimum loss to nutritional values [3]. HPP machines may operate at 100–1000 MPa pressure ranges [71]. Pasteurization, previously known as a heat‐based intervention, has been redefined, and HPP is now included in the definition of pasteurization as a nonthermal approach [72].

In an experiment to determine the sensitivity of *C. sakazakii* isolates to hydrostatic pressure, C. *sakazakii* strains were exposed to an increased hydrostatic pressure range between 200 and 600 MPa for 0 and 10 min in chicken soup, orange juice, rehydrated powdered milk, and vegetable soup. At 500 MPa, the most pressure‐resistant strain of *Cronobacter* showed roughly three log decreases in all food. The findings demonstrated that applying increased hydrostatic pressure to biotic surfaces could help eradicate the pathogen [73].

## CONCLUSION

The prevalence of *C. sakazakii* in food and environmental isolates, particularly infant foods, is a public health challenge in Africa. Still, there is evidently underreporting or underdiagnosis of this infection. The sporadic documentation of *Cronobacter* infection cases worldwide, particularly in Africa, means that Africa's disease surveillance systems need to do more to detect *Cronobacter* spp. alongside other foods, environmental, and medical disease sources.

There is a need to standardize control measures to prevent food contamination, introduce environmental monitoring systems, and strict adherence to thermal and nonthermal food processing and packaging controls.

## AUTHOR CONTRIBUTIONS

Ifeanyi Michael Mazi developed the concept for this review. Ifeanyi Michael Mazi, Helen Onyeaka, and Nnabueze Darlington Nnaji wrote the first draft of the manuscript. Helen Onyeaka and Ifeanyi Michael Mazi proofread and edited the language. Ifeanyi Michael Mazi revised the manuscript based on feedback from Helen Onyeaka. Helen Onyeaka supervised the project and critically revised the final manuscript. All authors contributed to the manuscript and approved the submitted version.

## CONFLICT OF INTEREST

We declare that we do not have any conflict of interest in publishing this manuscript.

## CONSENT FOR PUBLICATION

All authors consent to the publication of the article.

## ETHICS STATEMENT

This research did not contain any studies involving animal or human participants, nor did it take place on any private or protected areas.

## Data Availability

Data sharing not applicable to this article as no datasets were generated or analysed during the current study.
